# When Time Matters in Aortic Stenosis: Can Transcatheter Aortic Valve Replacement Make a Difference in Non-Elective Cases?

**DOI:** 10.31083/RCM49738

**Published:** 2026-07-24

**Authors:** Cecilia Villa Etchegoyen, Laurève Chollet, Mina Ahmed, Christine Bilauca, Juan M Farina, Milagros Pereyra, Isabel G. Scalia, Mahshad Razaghi, Fatmaelzahraa E. Abdelfattah, Kamal Awad, Abdelrahman Hafez, Sherif Ahmed, Chadi Ayoub, Said Alsidawi, John P. Sweeney, Steven J. Lester, Kwan S. Lee, David Fortuin, Kristen A. Sell-Dottin, Reza Arsanjani

**Affiliations:** ^1^Department of Cardiovascular and Thoracic Surgery, Mayo Clinic, Phoenix, AZ 85054, USA; ^2^Department of Cardiovascular Medicine, Mayo Clinic, Phoenix, AZ 85054, USA

**Keywords:** aortic valve stenosis, transcatheter aortic valve replacement, emergency treatment, mortality

## Abstract

**Background:**

The development of symptoms or left ventricular dysfunction in severe aortic stenosis (AS) is associated with high morbidity and mortality. While elective transcatheter aortic valve replacement (TAVR) is supported by robust randomized evidence, data on TAVR performed in non-elective settings (urgent, emergent, or salvage) remain limited and heterogeneous. In these settings, outcomes may be influenced more by baseline clinical severity than by procedural factors. This study aimed to systematically evaluate the safety and effectiveness of non-elective TAVR and to compare the associated outcomes with those of elective TAVR.

**Methods:**

This systematic review and meta-analysis were conducted in accordance with the Preferred Reporting Items for Systematic reviews and Meta-Analyses (PRISMA) guidelines and registered with PROSPERO. The PubMed, Embase, and Scopus databases were searched for studies published after 2002 evaluating urgent, emergent, or salvage TAVR in adult patients with severe AS. Eligible studies were required to include elective TAVR as a comparator. Outcomes included mortality and major procedural complications. Prespecified era-based analyses and sensitivity analyses excluding studies at critical risk of bias were performed. Risk of bias was assessed using the ROBINS-I tool.

**Results:**

A total of 17 observational studies published between 2015 and 2025 were included, comprising 215,141 patients. Compared with elective patients, those undergoing non-elective TAVR had more advanced heart failure, higher NYHA class, greater comorbidity burden, and higher surgical risk scores, whereas baseline echocardiographic severity of AS was similar. Pooled outcomes for non-elective TAVR showed in-hospital, 30-day, and 1-year mortality rates of 5.1%, 12.4%, and 26.7%, respectively. Non-elective TAVR was associated with higher mortality at all time points compared with elective procedures; meanwhile, rates of stroke, vascular complications, and permanent pacemaker implantation were similar, while major bleeding and acute kidney injury were more frequent. Era-based analyses showed a stable relative mortality risk over time.

**Conclusions:**

Although non-elective TAVR is associated with worse outcomes than elective procedures, these differences appear to be largely driven by baseline clinical severity rather than by the transcatheter intervention. In unstable patients with severe AS, non-elective TAVR remains the most effective definitive treatment option compared with balloon aortic valvuloplasty or conservative management.

**The PROSPERO Registration:**

CRD420251239620, https://www.crd.york.ac.uk/PROSPERO/view/CRD420251239620.

## 1. Introduction

Aortic stenosis (AS) is the most common valvular disease in adults, and its prevalence increases with age [1]. The condition usually worsens over time, and once symptoms or left-ventricular dysfunction appear, patients experience high morbidity, increased hospitalization, and reduced survival [2,3]. For this reason, timely and effective treatment is essential. Several treatment options exist for severe AS, including surgical aortic valve replacement (SAVR), transcatheter aortic valve replacement (TAVR), balloon aortic valvuloplasty (BAV), and conservative management for patients who are ineligible for invasive therapy.

Elective TAVR is well established and has demonstrated favorable safety and efficacy in stable patients, supported by strong randomized evidence [4,5]. In contrast, decision-making in non-elective settings (urgent, emergent, or salvage cases) is considerably more challenging. These patients often present with hemodynamic instability, organ dysfunction, or rapidly worsening symptoms. Procedural planning is difficult, and the available evidence remains limited. Most studies in this area are small, observational, and report outcomes inconsistently, making interpretation difficult [6]. Owing to these gaps, synthesizing all available data on TAVR in non-elective settings and comparing the associated outcomes with other options or clinical scenarios, such as BAV, conservative management, or elective TAVR, is important. However, the quantitative analysis in this study is limited to comparisons between non-elective and elective TAVR, whereas alternative strategies, such as BAV and conservative management, are discussed only in the clinical context. SAVR is excluded from this review to ensure the focus remains on transcatheter and non-surgical strategies.

This systematic review aims to evaluate the safety and effectiveness of TAVR performed in non-elective settings and to compare these outcomes with those of elective TAVR to support clinical decision-making in urgent and emergent scenarios.

## 2. Methods

This systematic review and meta-analysis were conducted in accordance with the Preferred Reporting Items for Systematic Reviews and Meta-Analyses (PRISMA) guidelines [7] and were registered in the PROSPERO database (CRD420251239620).

### 2.1 Search Strategy

A search for outcomes in urgent and emergent TAVI was performed using the Embase, PubMed, and Scopus databases. The Embase search strategy included the following terms: “transcatheter aortic valve replacement” OR “transcatheter aortic valve implantation” OR “TAVR” OR “TAVI” OR “transcatheter aortic valve” OR “percutaneous aortic valve replacement” AND “urgent” OR “emergency” OR “emergent” OR “urgency” OR “acute” OR “salvage”. The PubMed search strategy included the following terms: “Transcatheter Aortic Valve Replacement” [Mesh] OR “TAVR” OR “TAVI” OR “Transcatheter Aortic Valve Implantation” OR “Transcatheter aortic valve” OR “Percutaneous aortic valve replacement” AND “Emergencies” [Mesh] OR “Emergency Treatment” [Mesh] OR “Urgent” OR “Emergency” OR “Emergent” OR “Urgency” OR “Salvage”. The Scopus search strategy included the following terms: “transcatheter aortic valve replacement” OR “transcatheter aortic valve implantation” OR “TAVR” OR “TAVI” OR “transcatheter aortic valve” OR “percutaneous aortic valve replacement” AND “urgent” OR “emergency” OR “emergent” OR “urgency” OR “salvage”. All searches were limited to publications after 2002.

### 2.2 Eligibility Criteria

The population included patients aged over 18 years, of any sex, with severe AS undergoing TAVR in an urgent, emergent, or salvage context (cardiogenic shock, acute decompensated heart failure, or hemodynamic instability). Studies including mixed valvular populations were considered eligible only if most patients had AS or if outcomes were reported specifically for this subgroup. Studies primarily focused on isolated aortic regurgitation were excluded.

Since the terminology for procedure timing and clinical urgency was not uniform across studies, the terms urgent, emergent, salvage, and non-elective were considered conceptually related but not strictly standardized. This review used the term “non-elective TAVR” as the umbrella term for procedures performed outside an elective setting. To provide a general clinical framework, urgent procedures were defined as those performed shortly after decompensation due to life-threatening conditions or hemodynamic instability, often within hours. In contrast, emergent procedures were defined as those performed during acute admission for symptomatic decompensation, typically within the first 24–72 hours, without immediate life-threatening instability. However, these definitions were intended for conceptual purposes only and should not be interpreted as uniform criteria across studies. For data extraction and pooled analyses, patients were classified according to the original definitions reported in each study to preserve consistency with the source data while acknowledging the heterogeneity of study-level classifications. The intervention of interest in this study included urgent, emergent, or salvage transcatheter aortic valve implantation, regardless of valve type, generation, or vascular access route. Eligible studies were required to include elective TAVR as the comparator intervention, as the primary objective of this meta-analysis was to perform a quantitative comparison between non-elective and elective TAVR. Eligible study designs included randomized controlled trials (RCTs), prospective and retrospective cohort studies, case-control studies, national or multicenter registry analyses, and case series. Narrative reviews, editorials, opinion articles, case reports, systematic reviews, and protocols without results were excluded.

Studies were also included if the data reported at least one outcome of interest: (1) in-hospital all-cause mortality; (2) 30-day all-cause mortality; (3) 1-year all-cause mortality; (4) stroke or transient ischemic attack; (5) acute kidney injury; (6) major vascular complications; (7) major bleeding; (8) new permanent pacemaker implantation.

### 2.3 Study Selection and Data Extraction

All studies were imported into Covidence software (Veritas Health Innovation, Melbourne, Australia) for independent screening. Two reviewers (C.V.E. and C.B.) screened the studies according to the inclusion and exclusion criteria. Data extraction was independently performed by three reviewers (C.B., L.C., and M.A.), and any discrepancies were reviewed by C.V.E. to ensure accurate extraction. The following information were extracted: (1) demographics (age and sex); (2) baseline characteristics (hypertension, diabetes mellitus, atrial fibrillation, cerebrovascular disease (previous stroke or transient ischemic attack (TIA)), previous coronary artery bypass grafting (CABG), chronic lung disease, chronic kidney disease, peripheral vascular disease, prevalence of New York Heart Association (NYHA) functional class III/IV, and prevalence of cardiac arrest and cardiogenic shock); (3) baseline echocardiographic characteristics (mean gradient (mmHg); aortic valve (AV) area (cm^2^), Vmax (m/s); ejection fraction (%), and prevalence of moderate-to-severe mitral regurgitation); (4) procedural characteristics (access route (apical, aortic, axillary, or femoral), type of valve implanted (balloon-expandable vs. self-expandable), prevalence of valve-in-valve procedures, and BAV before TAVR).

### 2.4 Risk of Bias Assessment

To evaluate the quality of the selected studies, a risk-of-bias assessment was performed independently by two reviewers using the Cochrane Risk of Bias in Non-Randomized Studies of Interventions (ROBINS-I) tool [8]. All studies were evaluated for quality across 7 domains: bias due to confounding, bias in participant selection, bias in intervention classification, bias due to deviations from intended interventions, bias due to missing data, bias in outcome measurement, and bias in the selection of reported results. Using the ROBINS-I tool, the risk of bias for each domain was classified as unclear, low, or high. A consensus was reached by reviewer C.V.E for all quality assessment forms.

### 2.5 Statistical Analysis

The number of events and non-events for each outcome of interest extracted from the studies was entered into the dataset as raw counts. The pooled data included counts of events and non-events, as well as sample sizes for each intervention group. The number of cases in the two comparison groups and the odds ratios (ORs) for each study were calculated. To account for temporal changes in TAVR practice and outcomes, prespecified analyses were performed by stratifying studies by publication era, and pooled estimates were compared across eras. The significance of pooled OR estimates was tested using z-tests. Homogeneity of effect sizes across the studies was assessed using Cochrane’s Q test, and the magnitude of heterogeneity was quantified using the I^2^ statistic (inconsistency). Fixed-effect models were used for outcomes with low statistical heterogeneity (I^2^ < 25%). In contrast, random-effects models were applied when between-study heterogeneity was present or when a more conservative estimate was considered appropriate, given the anticipated clinical and methodological heterogeneity across studies. Begg’s funnel plots and Egger’s test were used to assess potential publication bias. Finally, a sensitivity analysis was performed to investigate the effect of individual studies on the overall meta-analysis; the meta-analysis was re-estimated by sequentially omitting each study to assess the robustness of the results. The 95% confidence intervals (CIs) were calculated, and two-tailed *p*-values < 0.05 were considered statistically significant. Statistical analyses were performed using Review Manager 5 (RevMan 5), version 5.4. (Copenhagen, The Cochrane Collaboration, 2020), and RStudio, version 2025.5.1.513 (Boston, MA, USA).

## 3. Results

### 3.1 Study Selection

**Fig. 1.**
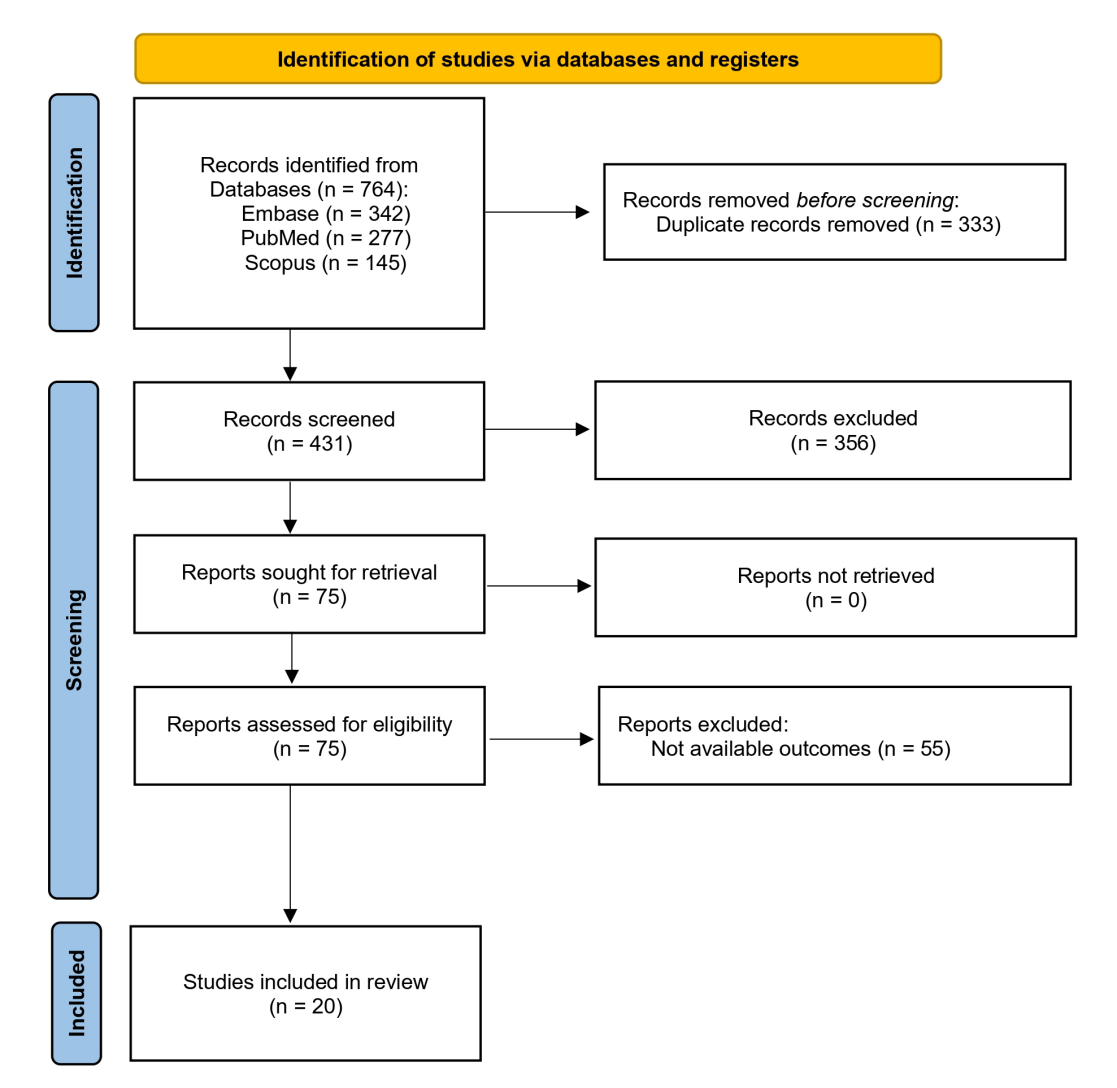
**Schematic of the Preferred Reporting Items for Systematic Reviews and Meta-Analyses (PRISMA) guidelines**. The numbers represent the records at each stage of the study selection process. Records were identified through database searches, and duplicates were removed before screening. Reports excluded at the eligibility stage did not meet the inclusion criteria because relevant outcome data were absent.

The search identified 764 records. After removing 333 duplicates, 431 titles and abstracts were screened. A total of 75 full-text articles were assessed for eligibility, and 20 studies were included in the final review [6,9,10,11,12,13,14,15,16,17,18,19,20,21,22,23,24,25,26,27]. Fig. 1 presents the PRISMA flowchart used to identify the included studies. Three studies were included in the qualitative synthesis but excluded from the meta-analysis due to a critical risk of bias [9,22,25].

### 3.2 Study Characteristics

The included studies were published between 2015 and 2025, with sample sizes ranging from 299 to 57,118 patients. Most studies were retrospective cohort studies, and all provided comparative data. Definitions of the exact timing of urgent, emergent, or salvage TAVR varied across studies; however, most described patients as presenting with hemodynamic instability, acute decompensation, cardiogenic shock, or rapidly worsening symptoms (Table 1, Ref. [6,9,10,11,12,13,14,15,16,17,18,19,20,21,22,23,24,25,26,27]).

**Table 1.** **Study characteristics**.

First author	Year	Country	Design	Total sample size	Comparison
Amgai [10]	2021	USA	Retrospective	40,385	• TAVR elective • TAVR urgent
Angerås [9]	2017	Sweden	Retrospective	405	• TAVR elective • TAVR urgent
Berkovitch [11]	2020	Israel	Retrospective	3599	• TAVR elective • TAVR urgent
Bianco [12]	2021	USA	Retrospective	1193	• TAVR elective • TAVR urgent
Castelo [13]	2023	Portugal	Prospective	299	• TAVR elective • TAVR urgent
Chen [15]	2020	USA	Retrospective	602	• TAVR elective • TAVR urgent
Elbadawi [14]	2020	USA	Retrospective	20,210	• TAVR elective • TAVR urgent
Enta [16]	2020	Japan	Retrospective	1613	• TAVR elective • TAVR urgent/emergent/salvage
Frerker [17]	2016	Germany	Retrospective	771	• TAVR elective • TAVR emergent
Ichibori [18]	2019	USA	Retrospective	476	• TAVR elective • TAVR urgent
Kabahizi [19]	2021	UK	Retrospective	1157	• TAVR elective • TAVR urgent/emergent/salvage
Kassis [20]	2020	USA	Retrospective	57,118	• TAVR elective • TAVR urgent
Kitahara [21]	2024	Japan	Retrospective	26,775	• TAVR elective • TAVR urgent/emergent
Kolte [6]	2018	USA	Retrospective	40,042	• TAVR elective • TAVR urgent/emergent
Landes [22]	2016	Israel	Retrospective	369	• TAVR elective • TAVR urgent
Lux [23]	2021	Netherlands	Retrospective	631	• TAVR elective • TAVR urgent
Oettinger [24]	2022	Germany	Retrospective	17,295	• TAVR elective • TAVR emergent
Saleh [25]	2025	USA	Retrospective	1216	• TAVR elective • TAVR emergent
Slade [26]	2023	USA	Retrospective	1564	• TAVR elective • TAVR urgent/emergent
Zhou [27]	2025	Australia	Prospective	1411	• TAVR elective • TAVR urgent

TAVR, transcatheter aortic valve replacement; USA, United States of America; UK, United Kingdom.

### 3.3 Patient Characteristics

Baseline characteristics of the study patients are summarized in Table 2 (Ref. [6,9,10,11,12,13,14,15,16,17,18,19,20,21,22,23,24,25,26,27]). Across the included studies, baseline clinical characteristics were generally comparable between elective and non-elective TAVR patients. Mean age ranged from approximately 78 to 84 years in both groups, and most studies reported no significant age difference. Females accounted for around 40%–55% of the population. Non-elective TAVR patients demonstrated a higher prevalence of advanced heart failure features, with NYHA class III–IV reported in approximately 70%–90% of patients, compared with 50%–70% in elective cases. Chronic kidney disease and chronic lung disease were more common in the non-elective group, with reported rates of approximately 30%–45% and 25%–40%, respectively. When surgical risk scores were available, non-elective patients had higher mean Society of Thoracic Surgeons (STS) or EuroSCORE values than elective patients, generally by 2–5 points.

**Table 2.** **Baseline demographic and clinical characteristics of patients undergoing elective versus non-elective transcatheter aortic valve replacement (TAVR)**.

	Age (y)		Female (%)		Diabetes mellitus (%)		Hypertension (%)		Atrial fibrillation (%)		Cardiovascular disease (%)		Chronic lung disease (%)		Chronic kidney disease (%)		Prior CABG (%)		PVD (%)		NYHA functional class III/IV (%)		STS or EuroSCORE (%)		Cardiac arrest (%)		Cardiogenic shock (%)	
First author	Elective TAVR	Non-elective TAVR	Elective TAVR	Non-elective TAVR	Elective TAVR	Non-elective TAVR	Elective TAVR	Non-elective TAVR	Elective TAVR	Non-elective TAVR	Elective TAVR	Non-elective TAVR	Elective TAVR	Non-elective TAVR	Elective TAVR	Non-elective TAVR	Elective TAVR	Non-elective TAVR	Elective TAVR	Non-elective TAVR	Elective TAVR	Non-elective TAVR	Elective TAVR	Non-elective TAVR	Elective TAVR	Non-elective TAVR	Elective TAVR	Non-elective TAVR
Amgai 2021 [10]	80.7 ± 8.25	79.42 ± 9.98	45.9	44.38	36.00	38.81	36.00	39.85	39.00	44.25	13.10	11.48	9.00	14.17	31.18	38.90	20.54	17.87	14.62	14.09	NA	NA	NA	NA	NA	NA	NA	NA
Angerås 2017 [9]	NA	NA	NA	NA	NA	NA	NA	NA	37.00	51.00	NA	NA	NA	NA	NA	NA	NA	NA	NA	NA	NA	NA	NA	NA	NA	NA	NA	NA
Berkovitch 2020 [11]	NA	NA	NA	NA	NA	NA	NA	NA	NA	NA	NA	NA	NA	NA	NA	NA	NA	NA	NA	NA	NA	NA	NA	NA	NA	NA	NA	NA
Bianco 2021 [12]	83 (78–87)	82 (75–87)	51.2	47.00	40.90	44.10	89.20	89.10	41.00	48.60	23.50	22.70	8.10	14.20	9.40	17.40	25.90	29.20	36.50	33.60	71.90	93.50	8.08 ± 10.13	2.94 ± 3.87	NA	NA	0	1.20
Castelo 2023 [13]	82 ± 7	82 ± 7	59.8	54.40	32.40	46.80	84.00	88.60	30.60	40.50	9.60	6.30	23.30	22.80	46.10	55.70	11.90	12.70	6.80	21.50	64.40	83.50	2.79	5.92	NA	NA	NA	NA
Chen 2020 [15]	86 (81–89)	86 (80–89)	41.4	42.45	34.13	38.85	89.42	92.09	47.08	47.48	9.94	12.23	37.58	35.97	NA	NA	23.54	26.62	25.92	20.14	81.21	86.33	5.17 (3.59–7.40)	6.8 (4.9–9.2)	NA	NA	NA	NA
Elbadawi 2020 [14]	80.6 ± 8.85	81.0 ± 9.05	47.1	50.30	36.50	34.40	78.80	77.60	NA	NA	NA	NA	33.90	33.60	37.50	41.70	22.10	18.80	29.30	29.30	NA	NA	NA	NA	NA	NA	NA	NA
Enta 2020 [16]	84.3 ± 5.0	84.9 ± 7.0	70.4	70.10	26.30	33.30	78.80	74.70	20.20	35.60	13.80	23.00	18.50	17.40	NA	NA	7.20	10.30	14.40	31.00	48.50	88.50	6.5 (4.6–9.2)	13.7 (8.2–21.0)	NA	NA	NA	NA
Frerker 2016 [17]	80 ± 7	78 ± 9	53.1	55.60	30.60	40.70	86.70	81.50	44.80	51.90	14.10	14.80	16.80	22.20	39.60	63.00	NA	NA	23.20	33.30	NA	NA	24.2 ± 16.1	60.% ± 21.1	NA	NA	NA	NA
Ichibori 2019 [18]	NA	NA	NA	NA	NA	NA	NA	NA	NA	NA	NA	NA	NA	NA	NA	NA	NA	NA	NA	NA	NA	NA	NA	NA	NA	5.10	NA	NA
Kabahizi 2021 [19]	82.0 ± 2.1	80.1 ± 7.0	46.7	42.4	18	21.9	NA	NA	19.1	23.00	7.00	4.90	20.3	18.6	12.40	38.80	14.3	12	23.4	23	NA	NA	6.5 (4.6–9.2)	13.7 (8.2–21.0)	NA	NA	NA	NA
Kassis 2020 [20]	83.1 ± 5.6	NA	NA	NA	33.60	35.70	80.90	80.10	NA	NA	NA	NA	NA	NA	34.50	43.30	NA	NA	NA	NA	NA	NA	NA	NA	NA	NA	NA	NA
Kitahara 2024 [21]	84 ± 8.2	81.3 ± 8.2	48.4	48.10	35.20	39.40	90.00	91.30	40.60	50.20	20.80	22.40	36.40	9.30	8.80	16.00	28.30	25.30	30.30	32.90	79.40	92.50		11.8 (7.6–17.9)	NA	NA	0	2.50
Kolte 2018 [6]	84 (78–88)	84 (78–88)	68.4	69.60	26.50	27.70	79.20	75.40	NA	NA	9.10	12.60	9.30	9.30	NA	NA	4.50	4.00	12.40	NA	24.70	74.20	6.0 (4.2–8.6)	13.3 (8.2–25.0)	NA	NA	NA	NA
Lux 2021 [23]	80 (76–84)	79 (73–85)	43.4	53.10	27.00	32.00	NA	NA	NA	NA	9.20	11.30	13.80	17.00	26.50	30.20	NA	NA	NA	NA	NA	NA	2.9 (1.7–4.5)	5.3 (3.4–10.9)	NA	NA	NA	NA
Landes 2016 [22]	NA	NA	NA	NA	NA	NA	NA	NA	NA	NA	NA	NA	NA	NA	NA	NA	NA	NA	NA	NA	NA	NA	NA	NA	NA	NA	NA	NA
Oettinger 2022 [24]	81.11 ± 6.08	81.24 ± 6.71	50.9	49.23	32.05	32.05	63.50	62.40	45.20	51.50	NA	NA	11.70	13.50	6.60	9.27	8.30	9.30	8.52	9.39	5.11	5.91	13.46 ± 9.84	13.46 ± 9.84	NA	NA	NA	NA
Saleh 2025 [25]	NA	NA	NA	NA	NA	NA	NA	NA	NA	NA	NA	NA	NA	NA	NA	NA	NA	NA	NA	NA	NA	NA	NA	NA	NA	NA	NA	NA
Slade 2023 [26]	81.0 ± 8.2	81.3 ± 8.2	43.9	34.60	39.60	39.50	NA	NA	38.60	44.40	9.60	12.30	46.70	55.60	41.70	42.00	NA	NA	29.80	32.10	65.60	72.80	NA	NA	NA	NA	NA	NA
Zhou 2025 [27]	82 (76–86)	80 (74–85)	41.7	32.20	30.40	39.60	79.50	70.10	31.40	32.30	8.20	11.80	11.80	4.80	37.80	48.80	10.20	7.10	9.60	9.50	31.40	65.40	3.2 (2–5)	3.9 (2.3–5.7)	NA	NA	NA	NA

Data are presented as the mean (mean ± standard deviation (SD)), median (interquartile range), or percentages (%), as reported in the original studies. NA indicates data not reported. TAVR, transcatheter aortic valve replacement; CVD, cardiovascular disease; CABG, coronary artery bypass grafting; PVD, peripheral vascular disease; NYHA, New York Heart Association; STS, Society of Thoracic Surgeons; EuroSCORE, European System for Cardiac Operative Risk Evaluation.

Preprocedural echocardiographic findings are presented in Table 3 (Ref. [6,9,10,11,12,13,14,15,16,17,18,19,20,21,22,23,24,25,26,27]). Baseline echocardiographic findings were largely similar between groups. The mean aortic valve area ranged from approximately 0.6 to 0.8 cm², and the mean transvalvular gradient ranged from 40 to 55 mmHg in both elective and non-elective patients. Left ventricular ejection fraction was preserved in most cohorts, with mean values of 50%–60%; however, reduced ejection fraction (<40%) was reported in 20%–30% of non-elective cases in some studies. Moderate-to-severe mitral regurgitation was more common in non-elective patients, affecting approximately 25%–40%, compared with 15%–25% in elective procedures.

**Table 3.** **Baseline echocardiographic parameters in patients undergoing elective versus non-elective TAVR**.

	Aortic valve area (cm^2^)		Mean gradient (mmHg)		Vmax (m/s)		EF (%)		Moderate-to-severe MR (%)	
First author	Elective TAVR	Non-elective TAVR	Elective TAVR	Non-elective TAVR	Elective TAVR	Non-elective TAVR	Elective TAVR	Non-elective TAVR	Elective TAVR	Non-elective TAVR
Amgai 2021 [10]	NA	NA	NA	NA	NA	NA	NA	NA	NA	NA
Angerås 2017 [9]	NA	NA	NA	NA	NA	NA	NA	NA	NA	NA
Berkovitch 2020 [11]	NA	NA	NA	NA	NA	NA	NA	NA	NA	NA
Bianco 2021 [12]	NA	NA	48.5 ± 13.4	49.3 ± 18.3	NA	NA	NA	NA	25.50	37.70
Castelo 2023 [13]	0.73 ± 0.20	0.66 ± 0.22	49 ± 14	48 ± 14	NA	NA	52 ± 12	45 ± 14	15.90	23.10
Chen 2020 [15]	0.6 (0.5–0.8)	0.6 (0.5–0.8)	44 (37–52)	46 (38–55)	4.1 (3.8–4.6)	4.1 (3.8–4.5)	57 (45–65)	50 (40–60)	NA	NA
Elbadawi 2020 [14]	NA	NA	NA	NA	NA	NA	NA	NA	NA	NA
Enta 2020 [16]	0.45 ± 0.12	0.41 ± 0.11	50.5 ± 18.0	50.2 ± 20.0	4.6 ± 0.78	4.5 ± 0.84	58.5 ± 11.9	47.9 ± 16.1	9.00	27.60
Frerker 2016 [17]	0.8 ± 0.3	0.7 ± 0.2	38.4 ± 15.6	35.1 ± 16.8	NA	NA	52.4 ± 13.2	39.5 ± 15.4	51.60	53.90
Ichibori 2019 [18]	NA	NA	NA	NA	NA	NA	NA	NA	NA	NA
Kabahizi 2021 [19]	NA	NA	NA	NA	NA	NA	NA	NA	NA	NA
Kassis 2020 [20]	NA	NA	NA	NA	NA	NA	NA	NA	NA	NA
Kitahara 2024 [21]	0.6 (0.5–0.8)	0.5 (0.4–0.7)	46.1 (38.0–59.4)	47.3 (36.0–63.0)	NA	NA	64 (57–70)	48 (34–63)	7.80	22.30
Kolte 2018 [6]	NA	NA	NA	NA	4.1 (3.8–4.5)	4.1 (3.8–4.5)	58.0 (50.0–65.0)	53.0 (37.0–60.0)	28.10	47.42
Landes 2016 [22]	NA	NA	NA	NA	NA	NA	NA	NA	NA	NA
Lux 2021 [23]	NA	NA	NA	NA	NA	NA	NA	NA	NA	NA
Oettinger 2022 [24]	NA	NA	NA	NA	NA	NA	NA	NA	NA	NA
Saleh 2025 [25]	NA	NA	NA	NA	NA	NA	NA	NA	NA	NA
Slade 2023 [26]	NA	NA	NA	NA	NA	NA	NA	NA	NA	NA
Zhou 2025 [27]	0.8 (0.7–0.9)	0.7 (0.6–0.9)	43 (37–51)	46 (37–57)	4.2 (3.9–4.5)	4.3 (3.9–4.7)	60 (55–65)	52 (40–60)	13.00	34 (28.3)

Data are presented as the mean (mean ± SD), median (interquartile range), or percentages (%), as reported in the original studies. NA indicates data not reported. TAVR, transcatheter aortic valve replacement; Vmax, peak aortic jet velocity; EF, ejection fraction; MR, mitral regurgitation.

Data on procedural characteristics are presented in Table 4 (Ref. [6,9,10,11,12,13,14,15,16,17,18,19,20,21,22,23,24,25,26,27]). These characteristics varied across studies. Transfemoral access was the predominant approach in both groups, used in approximately 70%–90% of elective cases and 60%–85% of non-elective cases. Alternative access routes, including transapical and transaortic approaches, were more commonly reported in non-elective TAVR, accounting for up to 15%–30% of procedures in some cohorts. Balloon-expandable and self-expandable valves were used with variable distribution; no consistent pattern was observed between groups. BAV before TAVR was performed more frequently in non-elective patients, occurring in approximately 20%–40% of cases, compared with 5%–15% in elective settings.

**Table 4.** **Procedural characteristics and device-related variables in elective versus non-elective TAVR**.

	Femoral access (%)		Aortic access (%)		Apical access (%)		Axillary access (%)		Balloon-expandable valve (%)		Self-expandable (%)		Valve-in-valve procedure (%)		BAV before TAVR (%)	
First author	Elective TAVR	Non-elective TAVR	Elective TAVR	Non-elective TAVR	Elective TAVR	Non-elective TAVR	Elective TAVR	Non-elective TAVR	Elective TAVR	Non-elective TAVR	Elective TAVR	Non-elective TAVR	Elective TAVR	Non-elective TAVR	Elective TAVR	Non-elective TAVR
Amgai 2021 [10]	NA	NA	NA	NA	NA	NA	NA	NA	NA	NA	NA	NA	NA	NA	NA	NA
Angerås 2017 [9]	NA	NA	NA	NA	NA	NA	NA	NA	NA	NA	NA	NA	NA	NA	NA	NA
Berkovitch 2020 [11]	NA	NA	NA	NA	NA	NA	NA	NA	NA	NA	NA	NA	NA	NA	NA	NA
Bianco 2021 [12]	79.50	82.20	0.20	0.40	4.90	0.40	11.20	12.60	38.20	41.70	61.80	58.30	3.40	11.70	3.80	4.50
Castelo 2023 [13]	95.00	87.30	NA	NA	NA	NA	NA	NA	NA	NA	NA	NA	NA	NA	3.70	5.10
Chen 2020 [15]	80.78	82.73	1.51	6.80	10.15	9.35	6.91	7.20	85.10	84.89	14.90	15.11	5.40	6.47	74.95	72.70
Elbadawi 2020 [14]	NA	NA	NA	NA	NA	NA	NA	NA	NA	NA	NA	NA	NA	NA	NA	NA
Enta 2020 [16]	NA	NA	NA	NA	NA	NA	NA	NA	90.90	94.20	90.90	57.00	NA	NA	NA	NA
Frerker 2016 [17]	78.40	92.60	0.50	0	12.80	0	8.30	7.40	48.50	40.70	50.00	59.30	NA	NA	NA	NA
Ichibori 2019 [18]	NA	NA	NA	NA	NA	NA	NA	NA	43.60	56.40	NA	NA	NA	NA	NA	NA
Kabahizi 2021 [19]	93.90	96.60	NA	NA	NA	NA	NA	NA	NA	NA	41.59	37.30	NA	NA	11.00	7.10
Kassis 2020 [20]	NA	NA	NA	NA	NA	NA	NA	NA	NA	NA	NA	NA	NA	NA	NA	NA
Kitahara 2024 [21]	88.70	90.80	1.10	0.70	7.20	4.20	NA	NA	75.10	71.90	24.80	24.80	24.70	28.20	NA	NA
Kolte 2018 [6]	78.70	75.40	4.60	6.50	13.50	14.70	0.30	0.10	74.30	75.60	24.60	24.60	4.40	9.70	15.50	1.36
Landes 2016 [22]	NA	NA	NA	NA	NA	NA	NA	NA	NA	NA	NA	NA	NA	NA	NA	NA
Lux 2021 [23]	13	41.20	NA	NA	9	2.80	NA	NA	NA	NA	NA	NA	NA	NA	NA	NA
Oettinger 2022 [24]	NA	NA	NA	NA	NA	NA	NA	NA	42.86	42.10	57.14	57.90	NA	NA	NA	NA
Saleh 2025 [25]	NA	NA	NA	NA	NA	NA	NA	NA	NA	NA	NA	NA	NA	NA	NA	NA
Slade 2023 [26]	92.50	91.40	NA	NA	NA	NA	NA	NA	NA	NA	NA	NA	NA	NA	3.60	12.30
Zhou 2025 [27]	98.50	98.40	NA	NA	NA	NA	NA	NA	43.80	NA	NA	NA	NA	NA	9.00	14.20

Data are presented as percentages (%), as reported in the original studies. NA indicates data not reported. TAVR, transcatheter aortic valve replacement; BAV, balloon aortic valvuloplasty.

### 3.4 Overall Outcomes of Non-Elective TAVR

Across all studies, patients undergoing urgent, emergent, or salvage TAVR had an in-hospital mortality rate of 5.1% (95% CI, 5.0–5.3), a 30-day mortality rate of 12.4% (95% CI, 12.0–12.9), and a 1-year mortality rate of 26.7% (95% CI, 25.9–27.5). Within 30 days of the procedure, stroke occurred in 1.3% of patients (95% CI, 1.2–1.5), major vascular complications in 2.1% (95% CI, 1.9–2.3), major bleeding in 26.5% (95% CI, 26.0–27.0), new pacemaker implantation in 5.6% (95% CI, 5.3–5.8), and acute kidney injury in 10.5% (95% CI, 10.1–10.8).

### 3.5 Non-Elective TAVR vs. Elective TAVR

**Fig. 2.**
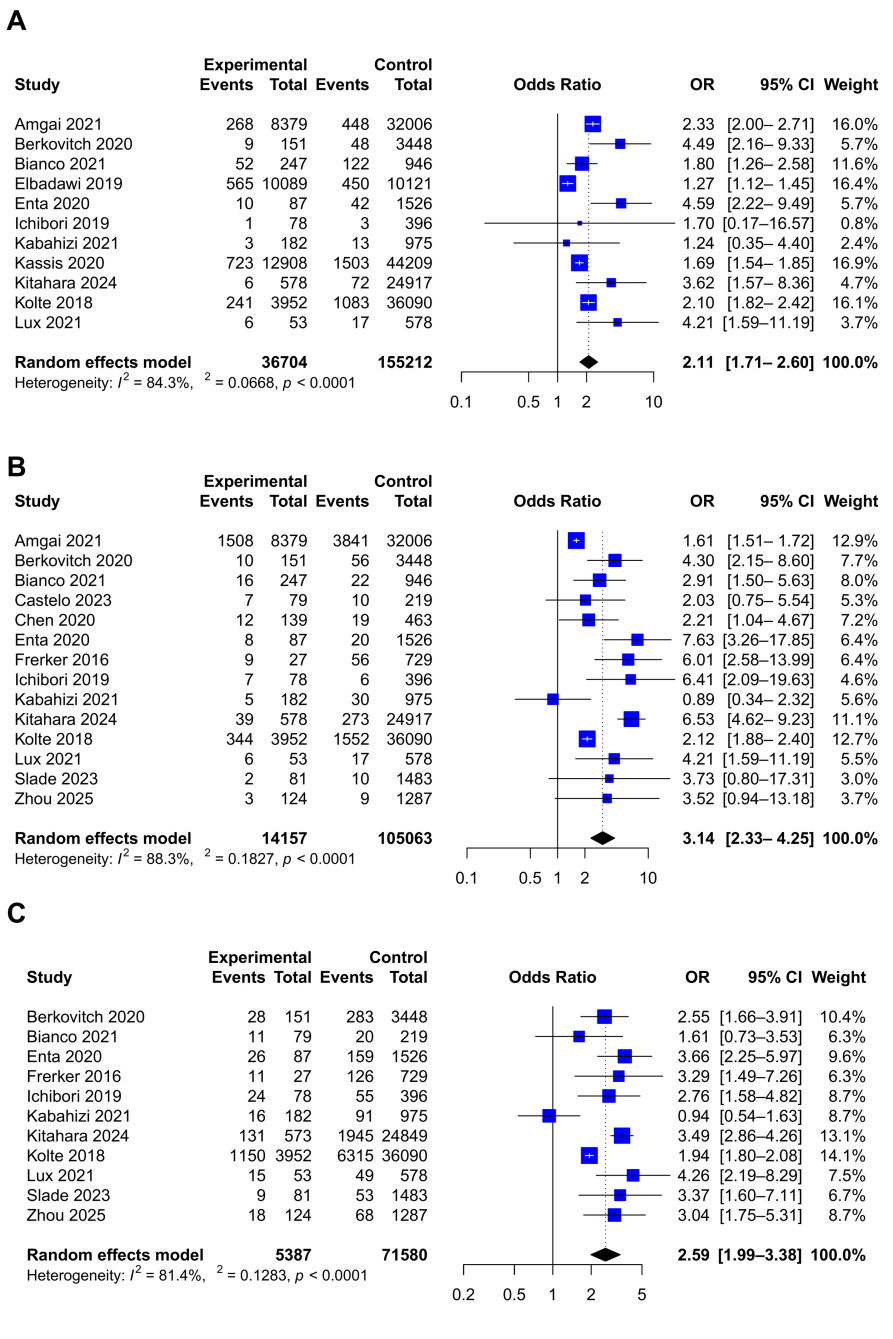
**Short and long-term mortality outcomes**. Forest plot of the (A) in-hospital, (B) 30-day, and (C) 1-year all-cause mortality rates between non-elective and elective transcatheter aortic valve replacement (TAVR), with odds ratios (ORs) and 95% confidence intervals (CIs). M–H, Mantel–Haenszel.

**Fig. 3.**
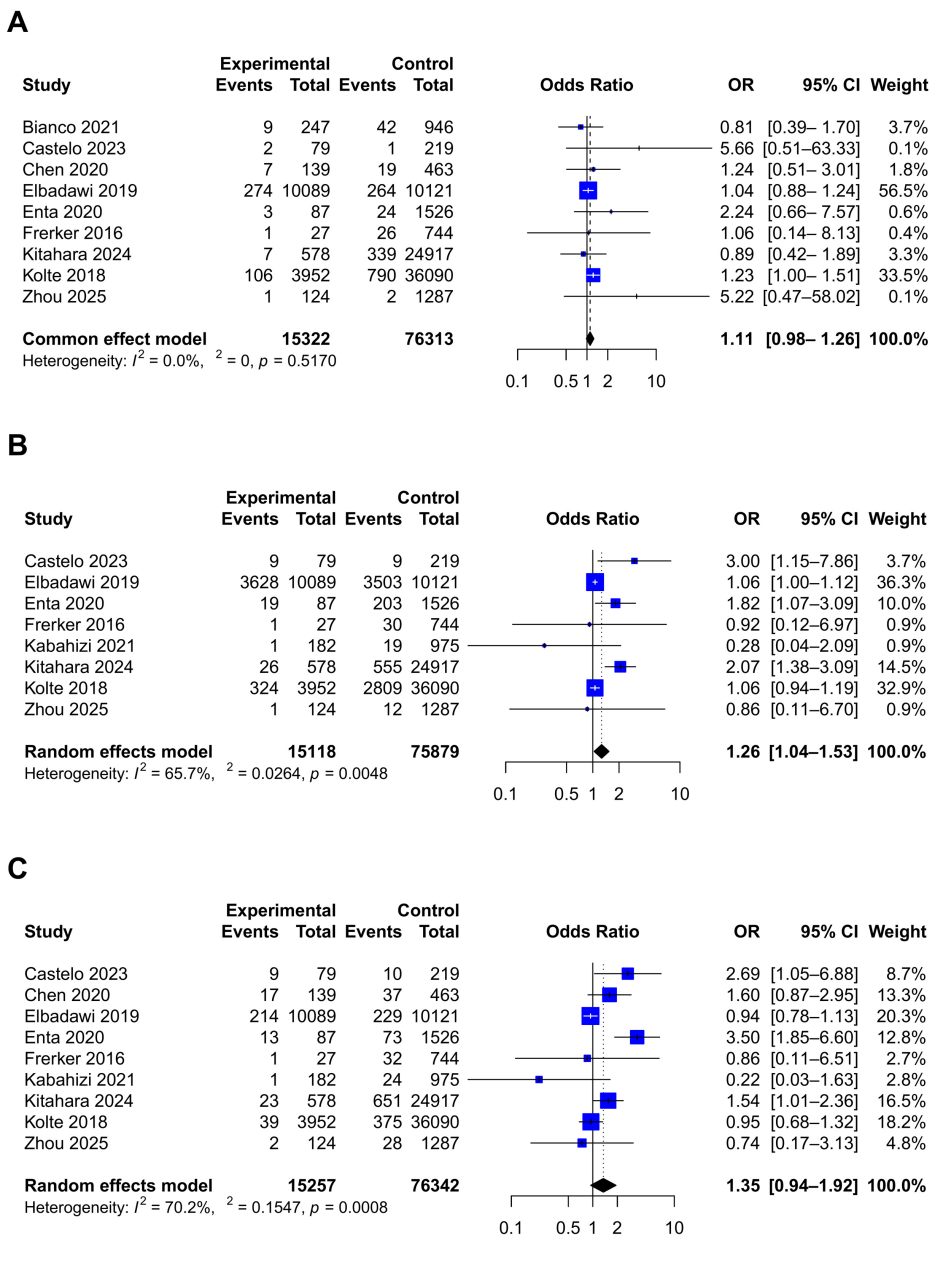
**Stroke, major bleeding, and major vascular complications outcomes**. Forest plot of outcomes for (A) stroke, (B) major bleeding, and (C) major vascular complications between non-elective and elective TAVR, with ORs and 95% CIs. M–H, Mantel–Haenszel.

**Fig. 4.**
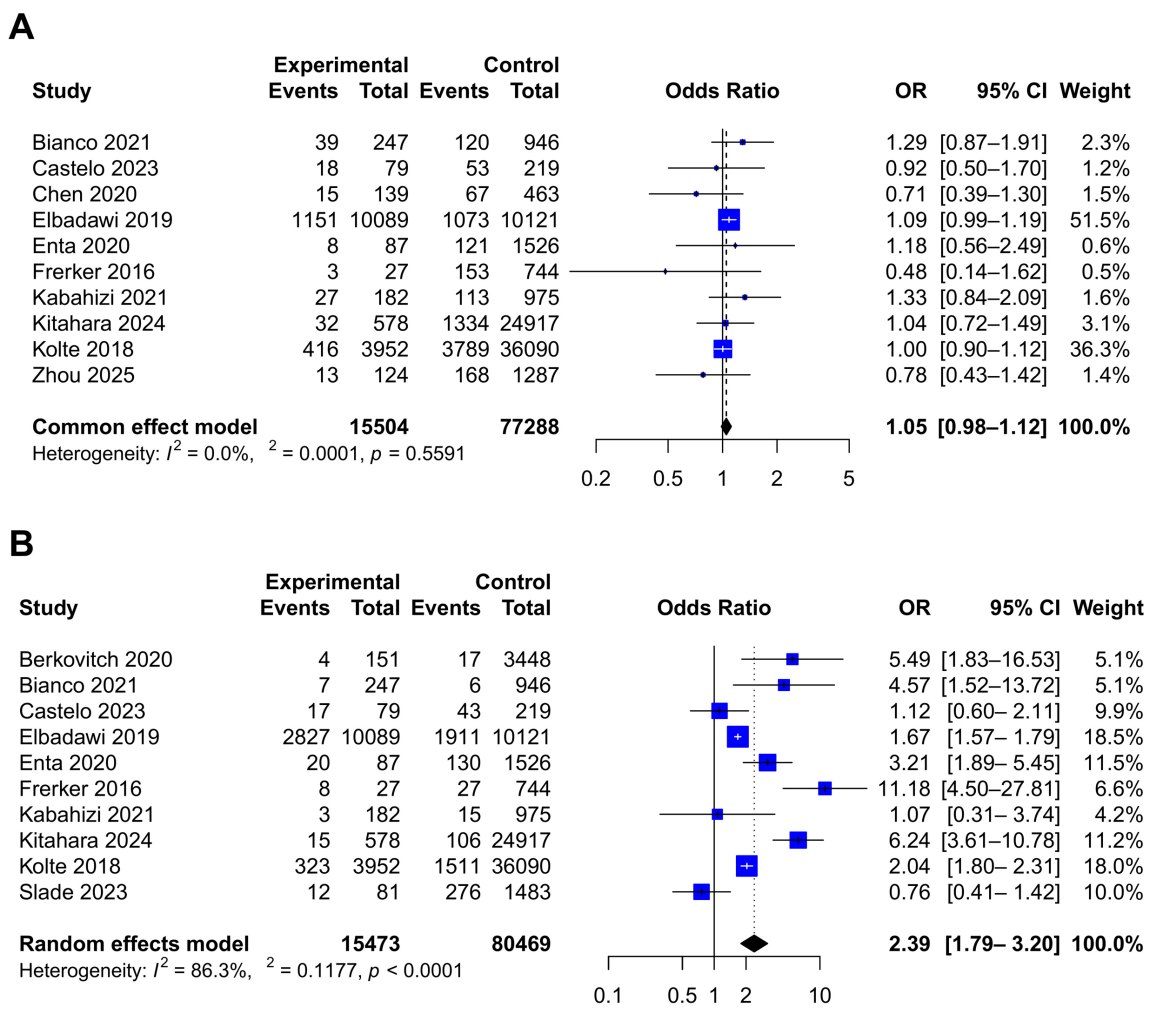
**Pacemaker implantation and acute kidney injury outcomes**. Forest plots of the (A) pacemaker implantations and (B) acute kidney injury outcomes between non-elective and elective TAVR, with ORs and 95% CIs. M–H, Mantel–Haenszel.

A total of 17 studies (n = 215,141) compared urgent, emergent, or salvage TAVR (non-elective TAVR) with elective TAVR. Non-elective TAVR was associated with higher in-hospital mortality (OR 2.11 (1.71–2.60)), 30-day all-cause mortality (OR 3.14 (2.33–4.25)), and 1-year mortality (OR 2.59 (1.99–3.38)) (Fig. 2). Compared with elective TAVR, non-elective TAVR was also associated with higher rates of stroke (OR 1.09 (0.96–1.23)) and major vascular complications (OR 1.35 (0.94–1.92)); however, these differences did not reach statistical significance. Major bleeding was significantly more frequent in the non-elective group (OR 1.25 (1.04–1.51)) (Fig. 3). No significant difference was observed in the rate of new pacemaker implantation between non-elective and elective TAVR (OR 1.05 (0.98–1.12)). In contrast, acute kidney injury occurred more frequently in patients undergoing non-elective TAVR (OR 2.39 (1.79–3.20)) (Fig. 4).

### 3.6 Eras Analysis

When outcomes were compared across eras (2015–2020 and 2021–2025), the higher risk associated with non-elective TAVR remained broadly similar. These findings suggest that, despite advances in TAVR technology and peri-procedural care, the excess mortality associated with non-elective TAVR has remained largely unchanged over time. In-hospital mortality was higher in the contemporary era, increasing from an OR of 1.99 (95% CI, 1.52–2.59) in 2015–2020 to 2.32 (95% CI, 1.89–2.83) in 2021–2025. In contrast, the associations for 30-day and 1-year mortality remained largely stable over time, with ORs of 3.22 (95% CI, 2.02–5.13) versus 2.77 (95% CI, 1.59–4.80) for 30-day mortality, and 2.35 (95% CI, 1.79–3.09) versus 2.44 (95% CI, 1.67–3.55) for 1-year mortality in the early and contemporary eras, respectively. Some complications showed modest improvement in the more recent era, such as stroke (OR 1.13 (0.99–1.28) vs. 0.78 (0.49–1.25)) and acute kidney injury (OR 3.85 (1.71–8.69) vs. 1.95 (1.09–3.50)). In contrast, major bleeding increased from an OR of 1.07 (0.99–1.17) to 1.79 (1.00–3.21) (**Supplementary Figs. 1–8**).

### 3.7 Sensitivity Analyses

Sensitivity analyses excluding studies with a critical risk of bias [9,22,25] did not meaningfully alter the magnitude or direction of the results for any evaluated outcome. Mortality outcomes and acute kidney injury retained statistically significant and clinically meaningful associations even after exclusion of high-risk studies. In contrast, bleeding, vascular complications, pacemaker implantation, and stroke outcomes did not show significant differences in any of the analyses.

### 3.8 Risk of Bias Assessment

Most of the included studies were observational and generally showed a moderate-to-serious risk of bias. The domains of greatest concern were confounding, mainly due to unadjusted baseline differences, and selection bias, as clinical instability often influenced the decision to perform urgent TAVR rather than the comparison strategy. We also identified issues related to deviations from intended interventions, especially in emergency settings, where strict protocol adherence was difficult, as well as missing outcome data in studies with incomplete follow-up or partially reported endpoints. In addition, some studies showed potential reporting bias when mortality at specific time points or procedural complications were not fully reported. A detailed summary of the risk-of-bias assessment across studies and domains is provided in **Supplementary Fig. 9**.

### 3.9 Publication Bias

Visual inspection of funnel plots did not suggest major asymmetry across outcomes. However, formal assessment using Egger’s test was limited by the relatively small number of studies for most endpoints; therefore, the results should be interpreted with caution. Funnel plots for the primary mortality outcomes (30-day and 1-year) are provided in **Supplementary Fig. 10**.

## 4. Discussion

### 4.1 Main Findings

This systematic review and meta-analysis provide a comprehensive overview of outcomes of TAVR performed in urgent, emergent, or salvage settings compared with elective procedures. The main finding is that non-elective TAVR is consistently associated with higher short- and long-term mortality and selected complications. However, these differences appear to be driven largely by baseline patient severity and clinical instability rather than by the TAVR procedure.

Patients undergoing non-elective TAVR represent a fundamentally different clinical population. As shown in the baseline characteristics, these patients more often presented with advanced heart failure, higher NYHA functional class, a greater burden of comorbidities, and higher surgical risk scores. These features are well-established predictors of adverse outcomes after any cardiovascular intervention [28] and strongly suggest that the observed excess mortality reflects the underlying disease state rather than procedural failure. Importantly, age and echocardiographic markers of AS severity were largely comparable between groups, indicating that the difference lies not in valve anatomy but in overall clinical status.

Procedural characteristics support the concept that TAVR remains technically feasible and broadly similar across settings. Transfemoral access remained the dominant approach even in non-elective cases, and the distribution of valve types did not show a consistent pattern favoring higher-risk devices. Notably, rates of stroke, vascular complications, and pacemaker implantation were comparable between non-elective and elective TAVR, reinforcing the notion that procedural safety is preserved even in urgent settings.

The era-based analyses provide further insight into the nature of the observed risk. Despite substantial advances in TAVR technology, operator experience, and peri-procedural care over the past decade, the relative excess mortality associated with non-elective TAVR remained stable across eras. This finding suggests that improvements in technique alone are insufficient to fully mitigate the prognostic impact of acute decompensation, cardiogenic shock, or rapidly progressive symptoms. In contrast, some complications, such as stroke and acute kidney injury, improved in the contemporary era, supporting the idea that procedural refinements and better supportive care can reduce selected risks, even if overall mortality remains largely determined by baseline severity. This observation underscores that procedural improvements alone may be insufficient to overcome the prognostic impact of acute clinical instability in non-elective patients.

### 4.2 Implications for Clinical Practice

The findings of this review have important implications for the management of patients with severe AS who present in an unstable or rapidly deteriorating condition. First, these findings indicate that TAVR is a reasonable treatment option in urgent, emergent, and salvage situations when surgery is not feasible or when delays would expose the patient to unacceptable risk. While prior studies suggest that non-elective TAVR may be associated with improved survival compared with BAV or conservative management, these comparisons are indirect and are presented for contextual interpretation only, as these treatment strategies were not formally compared within the pooled analyses of this study. In the PARTNER trial of inoperable patients, TAVR reduced 1-year all-cause mortality from approximately 50% with standard therapy, including BAV, to 30%, corresponding to an absolute risk reduction of nearly 20% [29]. Similarly, BAV alone has been associated with poor mid-term outcomes, with reported 30-day mortality rates often exceeding 27% [30], whereas contemporary series of urgent or emergent TAVR report 30-day mortality rates ranging from 2.4% to 11.32%, despite substantially higher baseline clinical risk [23,27].

The results highlight the need for rapid but structured assessment, including early involvement of the heart team, fast imaging protocols, and predefined institutional pathways for unstable patients. Centers performing TAVR should also be prepared for more frequent complications, including the need for hemodynamic and vascular management support.

Finally, because outcomes differ markedly among urgent, emergent, and salvage presentations, clinicians may use these categories to guide discussions with families and to set realistic expectations. Although non-elective TAVR carries a higher risk, the method remains the strategy with the best available outcomes for many critically ill patients with severe AS.

### 4.3 Limitations of the Included Studies

This study has several limitations. First, the included studies were retrospective and observational and showed important baseline differences between groups, thereby increasing the risk of reidual confounding even after adjustment. Although several studies reported adjusted estimates, the ability to fully account for differences in baseline clinical severity remains limited, particularly in high-risk and hemodynamically unstable patients. Factors such as cardiogenic shock, acute decompensated heart failure, and end-organ dysfunction are difficult to capture uniformly and may not be fully adjusted for in multivariable models. Thus, the higher mortality rates observed in non-elective TAVR likely reflect, at least in part, the underlying clinical instability of these patients rather than procedural risk alone. An additional limitation is the heterogeneity in study-level definitions of urgent, emergent, and salvage TAVR. Although we used non-elective TAVR as an umbrella term for conceptual consistency, pooled analyses were based on the original classification in each study, which may have introduced clinical heterogeneity across the included cohorts. Moreover, the non-elective population represents a spectrum of clinical acuity, with substantial differences in baseline severity, timing of intervention, and prognosis across urgent, emergent, and salvage presentations. Since the reporting was inconsistent and standardized definitions were lacking, more granular stratification was not feasible. In addition, key variables reflecting acute clinical severity, such as cardiogenic shock or cardiac arrest, were incompletely reported in many studies, further limiting detailed subgroup analyses.

Reporting was also inconsistent; indeed, some studies did not provide clear information on hemodynamic status, procedural details, or follow-up duration. Meanwhile, data on concomitant aortic regurgitation or mixed aortic valve disease were also reported inconsistently, limiting assessment of the impact of these conditions on outcomes. Other studies excluded patients who died before TAVR, which may underestimate the true risk associated with acute presentations. Finally, heterogeneity in outcome definitions for complications limits the precision of pooled estimates. Specifically, definitions of major bleeding were not uniform across studies: some used Valve Academic Research Consortium (VARC) criteria, others relied on registry-based or study-specific definitions, and reporting timeframes varied (in-hospital vs. 30-day). This variability may have contributed to the observed heterogeneity and relatively high rates of major bleeding.

Owing to these issues, the overall certainty of the evidence is low, and the conclusions should be interpreted with caution. These findings should be considered associative rather than causal, as the observed differences likely reflect baseline clinical severity rather than intrinsic procedural risk. More high-quality prospective studies are needed to define optimal strategies for managing non-elective severe AS.

## 5. Conclusions

This systematic review and meta-analysis shows that non-elective TAVR is associated with higher short- and long-term mortality than elective TAVR. However, these worse outcomes appear to be driven mainly by greater baseline clinical severity and hemodynamic instability rather than by the transcatheter procedure. Despite the higher risk profile, non-elective TAVR provides acceptable procedural safety and represents the most effective definitive treatment option compared with BAV or conservative management. These findings support the use of TAVR in urgent, emergent, and salvage scenarios, while highlighting the importance of early referral and optimized care pathways to improve outcomes. These data also support early heart-team activation and expedited TAVR pathways in unstable patients, as delaying intervention is unlikely to offset the poor prognosis associated with decompensation.

## Data Availability

Data that support the findings of this study may be available from the corresponding author upon reasonable request.
