# Supplementary material for: When Time Matters in Aortic Stenosis: Can Transcatheter Aortic Valve Replacement Make a Difference in Non-Elective Cases?
Source: Rev Cardiovasc Med. 2026 Jul 24;27(7):49738. doi: 10.31083/RCM49738 (PMC13419951; doi:10.31083/RCM49738)
Supplement: Supplementary file 1 [file 2153-8174-27-7-49738-s1.zip › Supplementary Material.docx]

**SUPPLEMENTARY DATA**

**SUPPLEMENTARY DATA**

**Supplementary Fig. 1. Eras analysis: In-hospital mortality**


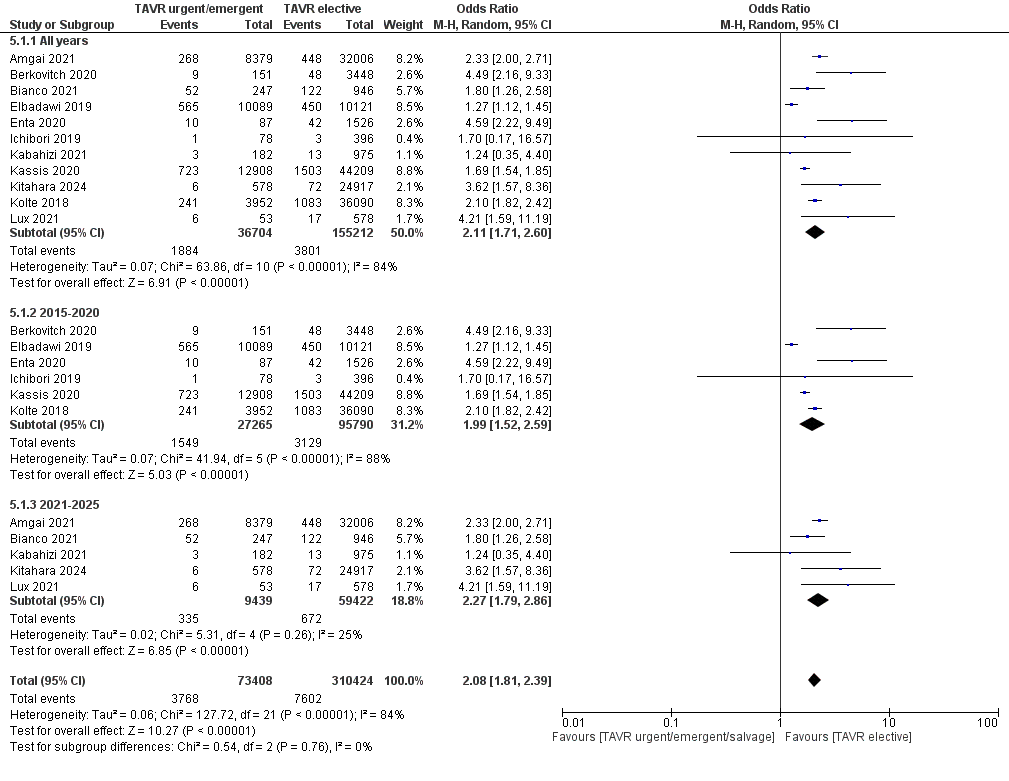


**Supplementary Fig. 2. Eras analysis: 30-days all-cause mortality**


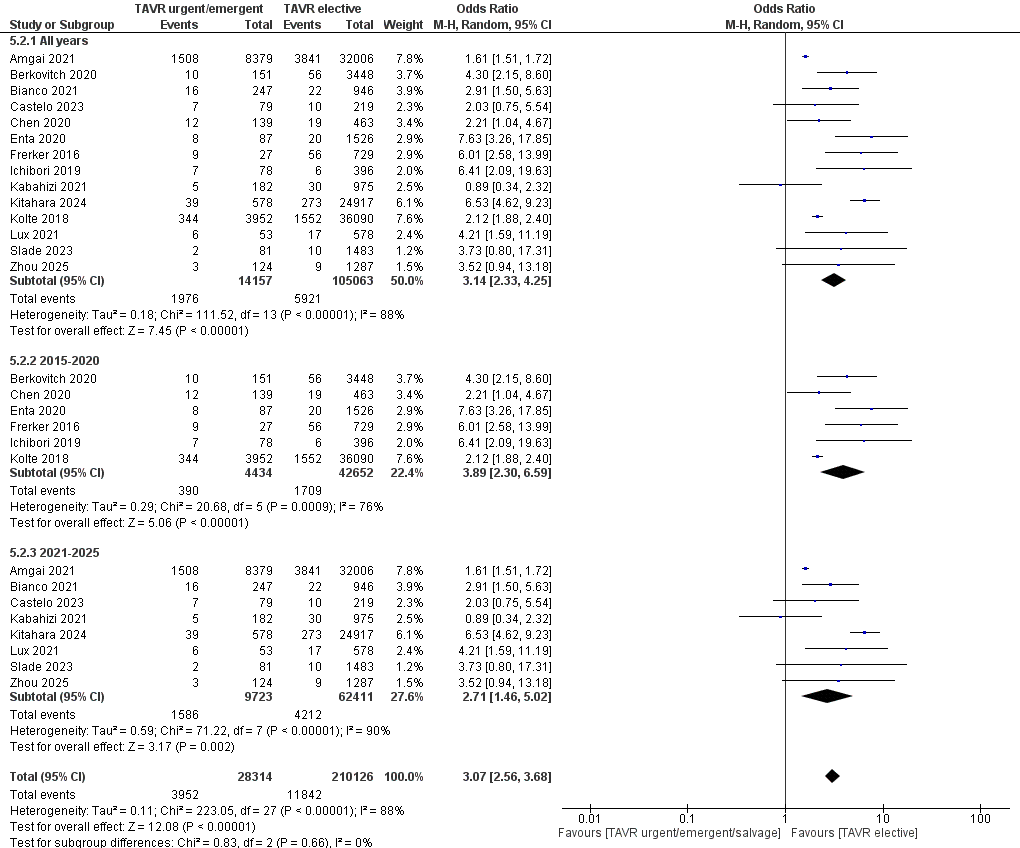


**Supplementary Fig. 3. Eras analysis: 1-year all-cause mortality**


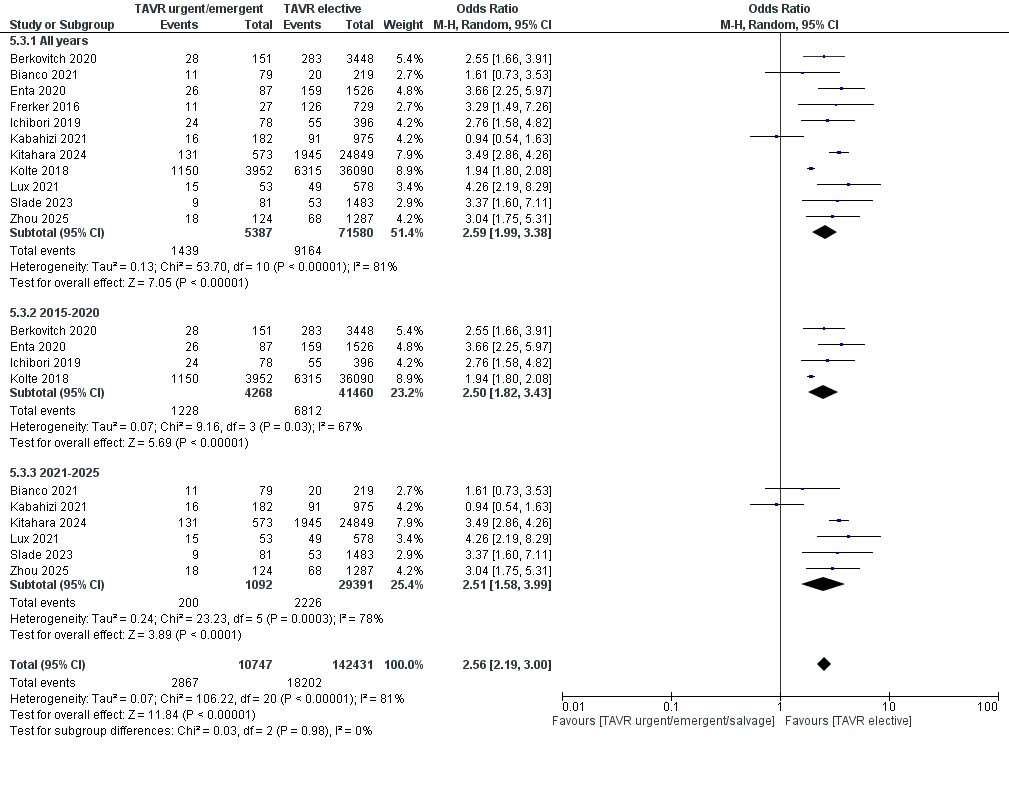


**Supplementary Fig. 4. Eras analysis: Major bleeding**


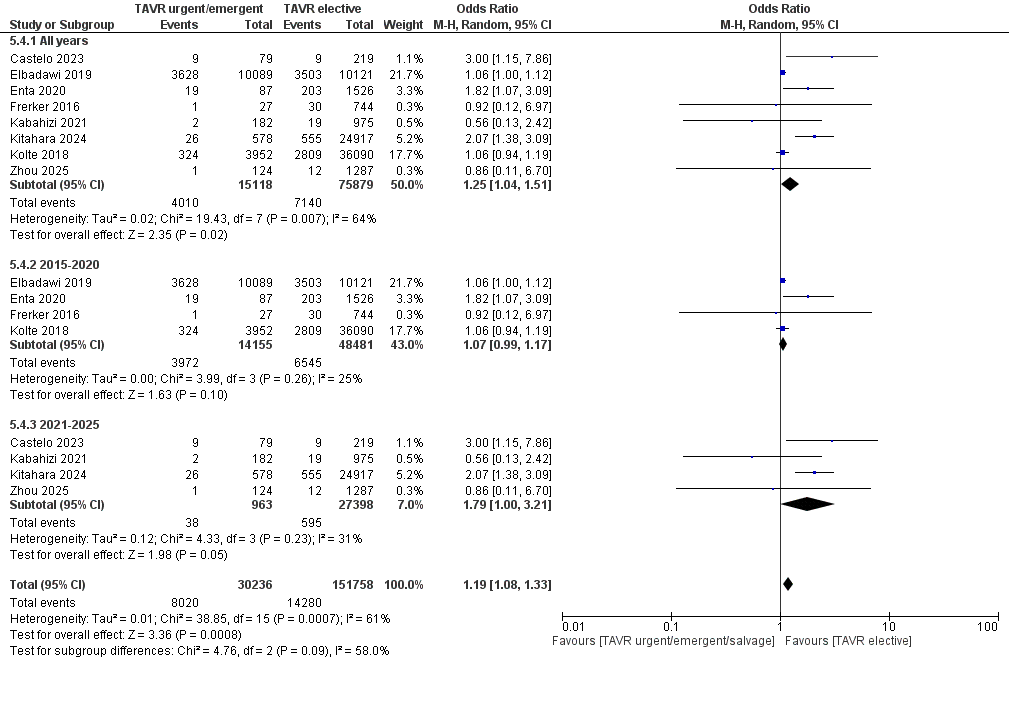


**Supplementary Fig. 5. Eras analysis: Major vascular complications**
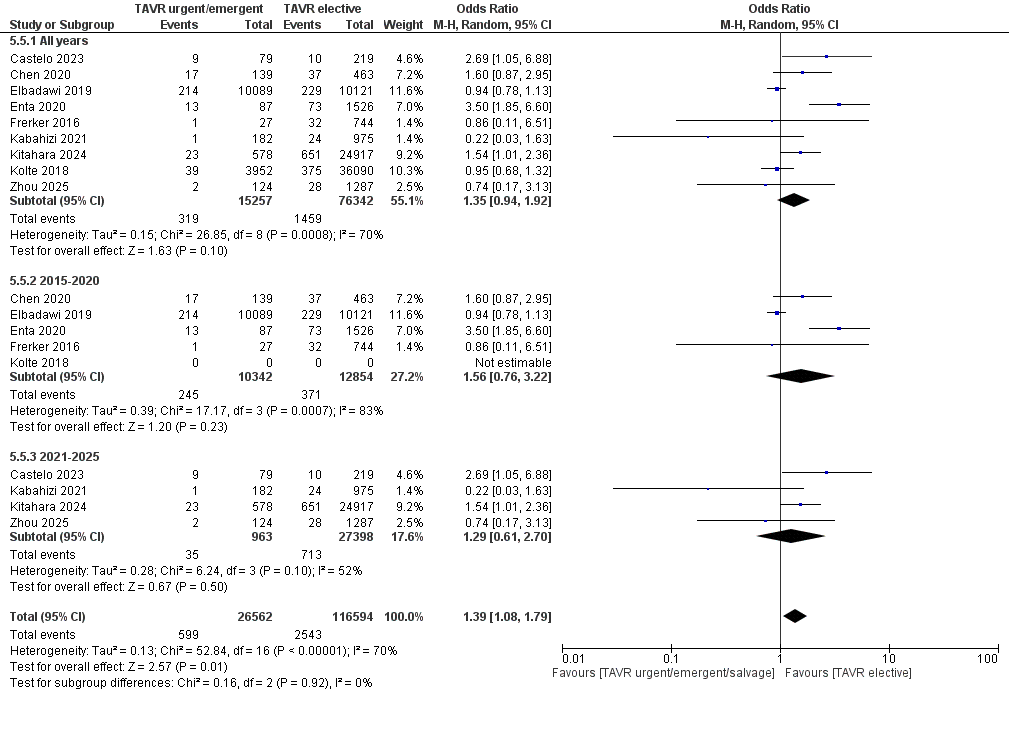


**Supplementary Fig. 6. Eras analysis: New pacemaker implantation**


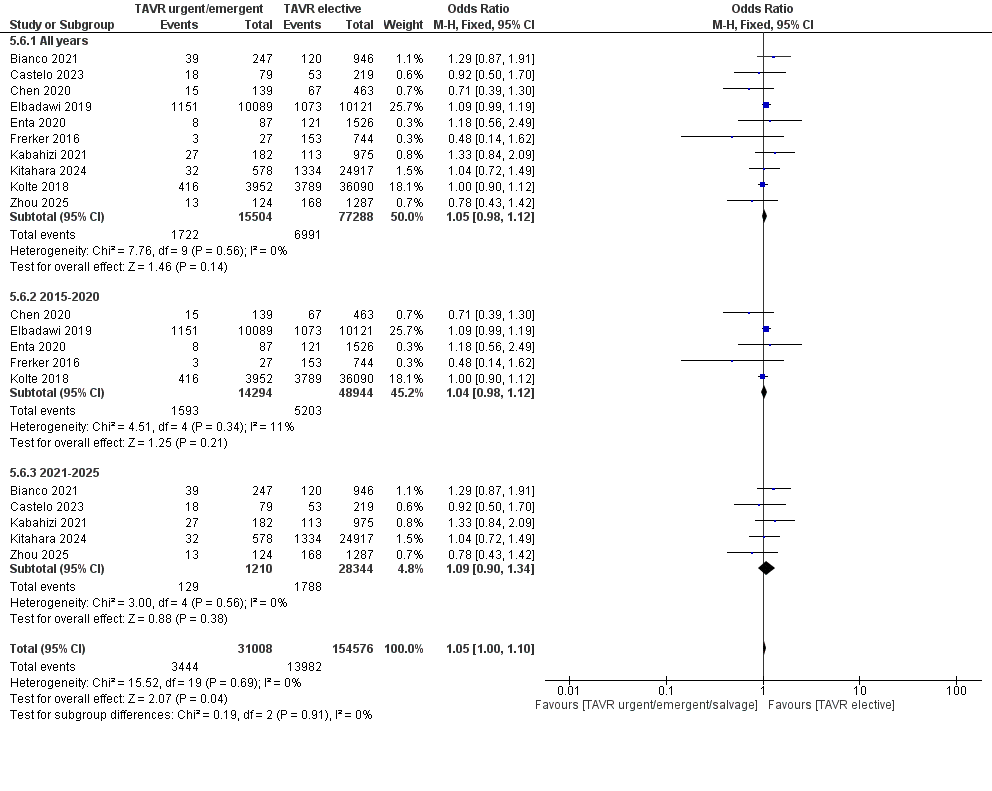


**Supplementary Fig. 7. Eras analysis: Stroke or TIA**


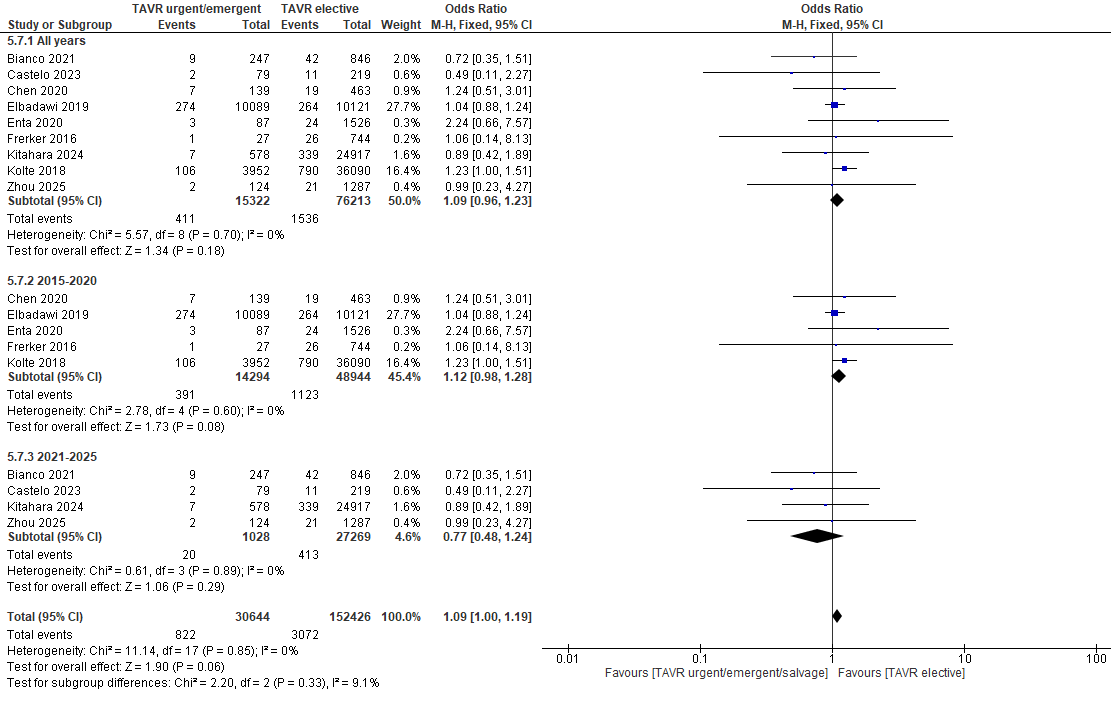


**Supplementary Fig. 8. Eras analysis: Acute kidney injury**


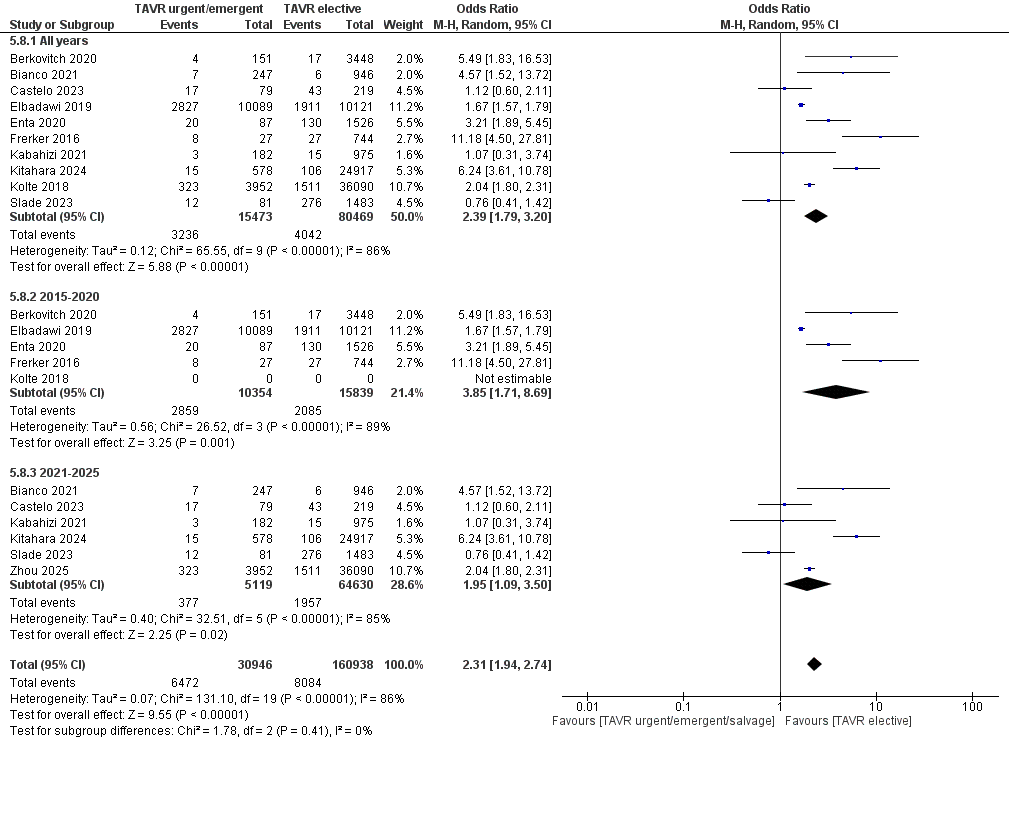


**Supplementary Fig. 9. Risk-of-bias assessment across included studies using the ROBINS-I tool.**


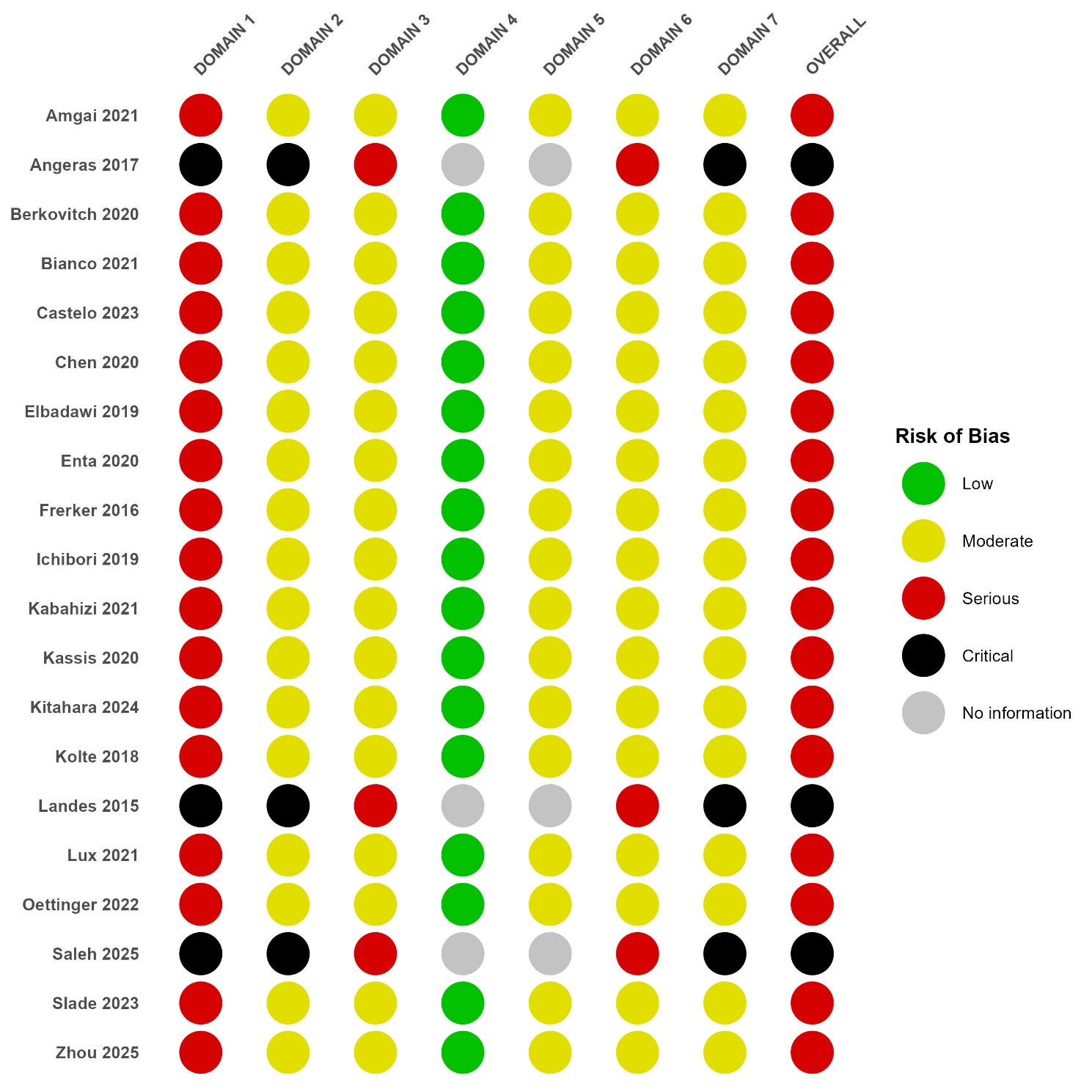


Circles represent study-level judgments for each bias domain and the overall risk of bias. Domain 1 (confounding); Domain 2 (selection of participants); Domain 3 (classification of interventions); Domain 4 (deviations from intended interventions); Domain 5 (missing data); Domain 6 (measurement of outcomes); Domain 7 (selection of reported results); Overall (overall risk of bias).

**Supplementary Fig. 10. Funnel plots for publication bias assessment across mortality outcomes**


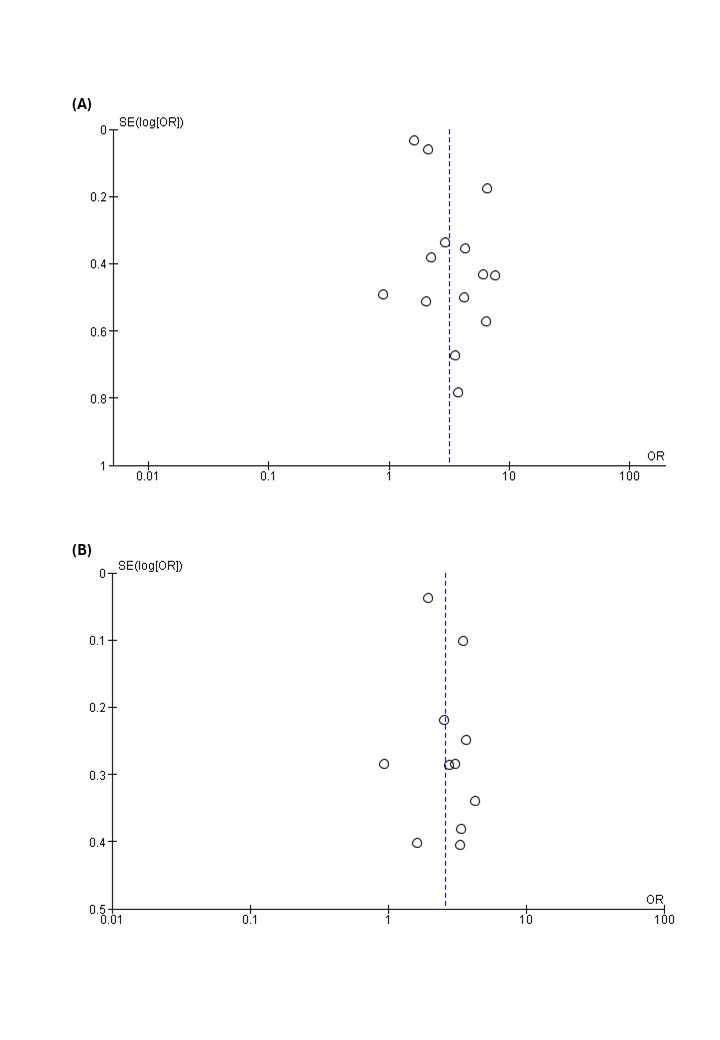


Funnel plots showing (A) 30-day all-cause mortality and (B) 1-year all-cause mortality across studies. The x-axis represents the odds ratio (OR) and the y-axis the standard error of the log odds ratio (SE [log OR]). The dashed vertical line indicates the pooled effect estimate. Visual inspection did not suggest major asymmetry; however, interpretation is limited by the relatively small number of studies per outcome. OR, odds ratio.
